# Childhood adversity predicts stronger premenstrual mood worsening, stress appraisal and cortisol decrease in women with Premenstrual Dysphoric Disorder

**DOI:** 10.3389/fendo.2023.1278531

**Published:** 2023-11-02

**Authors:** Sibel Nayman, Isabelle Florence Schricker, Iris Reinhard, Christine Kuehner

**Affiliations:** ^1^ Research Group Longitudinal and Intervention Research, Department of Psychiatry and Psychotherapy, Central Institute of Mental Health, Medical Faculty Mannheim, University of Heidelberg, Mannheim, Germany; ^2^ Department of Biostatistics, Central Institute of Mental Health, Medical Faculty Mannheim, University of Heidelberg, Mannheim, Germany

**Keywords:** Premenstrual Dysphoric Disorder, childhood adversity, stressful life events, cortisol, measurement burst, stress

## Abstract

**Background:**

Lifetime traumatic events are prevalent in women with Premenstrual Dysphoric Disorder (PMDD) and predict stronger premenstrual symptom intensities. Less is known about the unique effects of childhood adversity on PMDD. This study aims to investigate the menstrual cycle related course of mood, stress appraisal and cortisol activity over time and the effects of childhood adversity – by controlling for recent stressful life events – on the cyclicity of these outcomes.

**Methods:**

Fifty-two women with PMDD completed questionnaires on childhood adversity and stressful life events during the past 12 months. Momentary negative and positive affect, stress appraisal, and saliva-cortisol were assessed within an Ambulatory Assessment (AA) design over four consecutive days during both the follicular and the late luteal phase. This AA was repeated after five months, resulting in two measurement bursts.

**Results:**

Women with PMDD showed expected cycle related variations in mood and stress appraisal, whereby these effects weakened over time. No cortisol cyclicity was identified. Higher childhood adversity was linked to stronger increases in negative affect and stress appraisal, and stronger decreases in positive affect from the follicular toward the late luteal phase. Women with higher childhood adversity exhibited lower cortisol levels during the late luteal phase compared to the follicular phase whereas no such cyclicity was found in women with lower childhood adversity.

**Conclusion:**

Childhood adversity appears to show independent deteriorating effects on premenstrual mood worsening and stress appraisal in women with PMDD. The observed cortisol cyclicity in women with higher childhood adversity may point to different neuroendocrine subtypes of PMDD in relation to childhood trauma and requires further systematic research.

## 1 Introduction

Childhood adversity is a potential environmental risk factor for numerous mental and physical diseases in adulthood (1). Prolonged exposure to childhood adversity, including experiences of physical, sexual or emotional abuse as well as physical or emotional neglect, can cause chronic stress and result in neuroendocrine system malfunctions such as dysregulations of the Hypothalamic-Pituitary-Adrenal (HPA) axis (2).

Hantsoo & Epperson (3) suggest that childhood adversity makes women also vulnerable to reproductive mood disorders such as to perinatal (4) or perimenopausal depression (5). However, less is known about its role as a risk factor for ;Premenstrual Dysphoric Disorder (PMDD). PMDD is characterized by cyclical recurrences of impairing or distressing key affective symptoms and additional psychological and somatic symptoms during the late luteal phase of the menstrual cycle, with symptom remission after the onset of menstruation during the follicular phase (6). Cases with milder symptom intensities or lacking key affective symptoms with moderate distress are classified as Premenstrual Syndrome (PMS). In contrast to PMS, PMDD has been acknowledged as a separate diagnostic entity in the chapter of depressive disorders in DSM-5 (6) and as a gynecological disorder in ICD-11 (7). These differential classifications mirror the multifactorial etiology of PMDD, including psychosocial and (neuro-)endocrinological factors (8, 9). Regarding the latter, previous research consistently suggests that women with PMDD exhibit normal ovarian steroid levels, but show increased central nervous system sensitivity to these normal fluctuations of ovarian steroids and their neuroactive metabolites, especially allopregnanolone (ALLO) (8). In addition, there is initial evidence for altered HPA axis function in women with premenstrual disorders, such as lower basal and stress-reactive cortisol activity in women with PMS (10) and PMDD (11–14) as well as a delayed cortisol awakening response peak and a flattened diurnal cortisol slope across the menstrual cycle in women with PMDD compared to healthy controls (15). Two previous meta-analyses revealed menstrual cycle related variation of cortisol activity in healthy women, with lower cortisol levels during the luteal phase compared to the follicular phase (16, 17). In line with these meta-analyses, we identified respective menstrual cycle related cortisol cyclicity in healthy women, but not in women with PMDD (13). Given that cyclical ovarian steroid levels and their metabolites interact with the HPA axis, a history of chronic stress and adversity related HPA axis dysfunction may consequently contribute to the etiology and maintenance of PMDD and increase premenstrual symptom severity (cf. 9, 18).

Self-reported childhood adversity has been shown to be associated with higher premenstrual symptoms in adulthood in non-clinical samples (19, 20). Furthermore, in a prospective cohort study over 14 years, childhood adversity, particularly emotional and physical abuse, increased the risk of moderate-to-severe PMS in a sample of women who were free from PMS at baseline (21). Relatedly, in women with PMDD, a high prevalence (83%) of childhood adversity has been observed, with all adversity types (i.e., physical abuse, sexual abuse, emotional abuse, and neglect) being more common in women with PMDD compared to the general female population in Australia (22). Similarly, a higher percentage of early life trauma before the age of 18 was found in women with PMDD compared to healthy controls in a German sample (15).

However, previous research on PMDD has mainly investigated effects of traumatic or stressful events at any stage of life on premenstrual symptoms, without differing between childhood (prior to age 18) and adulthood adversity (after age 18). For example, a prospective cohort study over 42 months showed that traumatic events at any time up to baseline increased the odds of developing PMDD at follow-up (23). Similarly, in a cross-sectional study, lifetime trauma and post-traumatic stress disorder (PTSD) were independently associated with PMDD or with premenstrual symptoms (24), and in women with prospectively assessed cycle related mood disorders (PMDD and PMS), a lifetime history of abuse predicted a stronger intensity of premenstrual symptoms (18). In this context, Girdler et al. (12) showed that women with PMDD report more lifetime sexual and physical abuse as well as a younger age of first abuse compared to healthy controls. Less is known about the effect of childhood adversity on alterations in HPA axis functioning in PMDD. Hitherto, research has included only small subsamples of abused versus non-abused women with PMDD, thereby showing that these subgroups did not differ with regard to basal or stress-reactive plasma cortisol, while alterations in adrenergic physiology were observed in the abused subsample (12). Another study from the same group showed that regardless of PMDD status, abused women showed lower plasma cortisol at rest and during stress, whereas again adrenergic indicators, i.e., vascular resistance and blood pressure during rest and during stress, were increased only in abused women with PMDD (25). However, due to a lack of studies with larger samples, more research on the role of childhood adversity for HPA axis related alterations in women with PMDD is warranted. Since a majority of studies shows that both childhood adversity (cf. 26) and PMDD (cf. 27) result in blunted HPA axis activity in women, the possible role of childhood adversity as an aggravating factor for respective HPA axis dysregulation in PMDD remains to be resolved.

To sum up, according to the biological embedding model, especially stress exposure during developmental sensitive periods during childhood (prior to age 18) may lead to dysfunctional structural and functional neuroanatomical as well as neuroendocrine changes (28, 29) with long-lasting effects contributing to the development and maintenance of psychopathology. However, most previous studies in women with PMDD did not differ between childhood and adulthood adversities, and therefore the independent role of childhood adversity remains understudied.

### 1.1 Aims

The present study was designed to focus on childhood adversity by controlling for more recent stressful life events, and to investigate independent effects of childhood adversity on the cycle related course of mood, stress appraisal and cortisol activity in daily life in women with PMDD. First, the menstrual cycle related variation of mood, stress appraisal and cortisol activity was investigated together with its possible change over a five-months interval. Next, we hypothesized that childhood adversity would be associated with stronger increases in negative affect and stress appraisal, and stronger decreases in positive affect from the follicular to the late luteal phase. In addition, we investigated the possible impact of childhood adversity on cortisol levels and menstrual cycle related cortisol cyclicity. Due to the overall lack of research with larger samples in this area, these analyses were exploratory.

## 2 Methods

This study is part of a larger project and an extension of previous analyses on cycle related mood and cortisol in women with PMDD and healthy controls (13). The present study includes a second wave (burst) of Ambulatory Assessment (AA) for the subsample of women with PMDD, and the analyses of menstrual cycle related cyclicity as a function of childhood adversity in these women.

### 2.1 Participants

The present study consisted of two measurement bursts separated by an interval of five months (*M* = 20.61 weeks, *SD* = 2.99). Participants were recruited via online advertisement on the website of the Central Institute of Mental Health (CIMH) in Mannheim, Germany, and via social media and online PMDD support groups. Women were eligible for the study if they fulfilled the DSM-5 diagnostic criteria for PMDD, as assessed using the Structured Interview for PMDD (SCID-PMDD, see below; 30). A diagnosis of PMDD requires meeting at least five of eleven PMDD symptoms, including at least one affective symptom, in the majority of menstrual cycles of the preceeding 12 months. These symptoms must be associated with clinically significant distress or functional impairment and may not merely represent an exacerbation of another disorder (6).

Further inclusion criteria included ages between 20 and 42 years, regular menstrual cycles (fluctuations < 5 days), an average cycle length of > 22 and < 34 days, and a BMI of > 18 and < 35. Exclusion criteria comprised a) pregnancy or breastfeeding during the last six months, b) intake of hormonal contraceptives, psychotropic drugs (e.g., Selective Serotonin Intake Inhibitors) and other medications with lasting effects on cortisol activity during the last six months, c) gynecological disorders (i.e., endometriosis, hysterectomy, oophorectomy), d) current depressive, generalized anxiety, eating and substance use disorder, e) lifetime history of bipolar disorder and psychosis. Shift working with regular late or night shifts and regular intensive exercising (> one hour/day) were additional exclusion criteria due to possible effects on cortisol activity. Initially, 60 women with PMDD were recruited at burst 1. Thereof, 52 completed both burst 1 and burst 2 and were included into the current measurement burst analyses. Four individuals provided information for their decision not to complete the AA at burst 2 (i.e., stay abroad, experienced burden at burst 1, lack of interest), whereas four further participants did not respond to our e-mails regarding the appointment proposals at burst 2. The description of the total sample at burst 1, including a comparison sample of healthy controls, can be found in (13).

### 2.2 Procedure

Both bursts consisted of a baseline session and an AA-phase during the follicular and the late luteal phase of the menstrual cycle. If the inclusion criteria were preliminarily met during a telephone screening session, participants were invited to a baseline session. At burst 1, baseline sessions took place either in person at the CIMH (*n* = 14) or, due to Covid-19 related contact restrictions, online via RedConnect (Red Medical Systems GmbH, Munich, Germany; *n* = 38). Given the high feasibility of the online format for the baseline sessions, all baseline sessions at burst 2 were also held online via RedConnect (*n* = 52). The study protocol was approved by the ethics committee of the Medical Faculty Mannheim, Heidelberg University. All participants provided written informed consent and were compensated with 240€ for the completion of both measurement bursts.

#### 2.2.1 Baseline sessions

During the baseline sessions at burst 1 and burst 2, structured clinical interviews were administered and additional demographic, clinical and cycle related characteristics were assessed.

##### 2.2.1.1 Psychopathology

For the assessment of PMDD criteria, we administered the Structured Interview for DSM-IV-defined PMDD (SCID-PMDD, interrater reliability κ = 0.96) (30), which was adapted for DSM-5 criteria (cf. 31). The SCID-PMDD covers all eleven symptom criteria of PMDD and assesses their presence, their onset- and offset-time during the cycle, as well as their frequency during the last 12 months. In addition, the SCID-PMDD checks for the criterion of relational, occupational, and recreational impairment or distress and the exclusion criterion of a mere exacerbation of symptoms of another disorder (cf. 32). For the current study, we decided against the additional administration of prospective symptom ratings as required by DSM-5 (6) in order not to overburden participants within the extensive AA-design with two bursts. Diagnostic exclusion criteria regarding current and lifetime psychiatric comorbidities at burst 1 were checked by administering the Structured Clinical Interview for DSM-IV-TR Axis I (SCID-I) (33). All interviews were performed by trained research psychologists.

##### 2.2.1.2 Childhood Adversity

Childhood adversity was assessed at burst 1 using the self-report Childhood Trauma Questionnaire (CTQ) (34) (German version: 35), which consists of 25 items measuring physical, sexual, and emotional abuse as well as physical and emotional neglect. Each subscale contains five items on a five-point Likert-scale ranging from 1 (never true) to 5 (very often true). For the current analyses, these subscales were summed up, resulting in a CTQ total score range from 29 to 85. Cronbach’s α of the total score amounted to α = .919 in the present study.

##### 2.2.1.3 Recent stressful life events

Stressful life events during the last 12 months before burst 1 were assessed using a list of 12 stressful life events (36), which were adapted from the List of Threatening Experiences (37) and the Schedule of Recent Events (37, 38) by Berntson et al. (36). The assessed events fall into the domains of health (e.g., “Did any of your family members or close friends die?”, social (e.g., “Did you have serious problems with a neighbour, friend or relative?”, job (e.g., “Were you fired or laid off from a job?”, and legal (e.g., “Did you or a family member have trouble with the police, get arrested or get sent to jail?”) (36). At burst 1, participants were asked to indicate whether or not they had experienced some of the listed 12 stressful life events (0 = no; 1 = yes). For the statistical analyses, we computed a stressful life events score by summing up the number of indicated events (cf. 36).

##### 2.2.1.4 Premenstrual Symptoms

Participants were asked to complete the German version of the 19-item *Premenstrual Symptom Screening Tool* (PSST) (39)(German version: 40) after the last AA-prompt of the 4-day AA-period during both the follicular and the late luteal phase. The original instruction, in which the items refer to the premenstrual phase, was modified such that participants were asked to rate their symptoms during the last four days (i.e., during the respective AA-period) on a 4-point Likert-scale (0 = not at all, 1 = mild, 2 = moderate, 3 = severe). In the present study, the internal consistency was high in both cycle phases and bursts (follicular phase: Cronbach’s α_Burst-1_ = .931, Cronbach’s α_Burst-2_ = .955; late luteal phase: Cronbach’s α_Burst-1_ = .894, Cronbach’s α_Burst-2_ = .905).

#### 2.2.2 Ambulatory Assessment (AA)

The AA protocol was identical for burst 1 and burst 2. During both bursts, the AA took place on four consecutive days during both the follicular and the late luteal phase, resulting in eight assessment days per burst. The AA was carried out using smartphones (Motorola e^6s^, Motorola g^8^, Nokia 4.2) with the software movisensXS, Version 1.5.12 (movisens GmbH, Karlsruhe, Germany, 2020).

Individual cycle calendars presenting scheduled AA days during the follicular and the late luteal phase were prepared based on participants’ self-reported average cycle length and the onset date of the last menses. The AA phase during the follicular phase was identified as days six to nine of the menstrual cycle with the day of menses onset representing day 1 of the cycle (cycle-day of AA-start: *M*_Burst-1 = _6.19, *SD*_Burst-1 = _0.53; *M*_Burst-2 = _6.28, *SD*_Burst-2 = _0.78). The late-luteal phase was defined as days −4 to −1 (cycle-day of AA-start: *M*_Burst-1 = _25.56, *SD*_Burst-1 = _2.55; *M*_Burst-2 = _24.51, *SD*_Burst-2 = _1.98), counting backward from the last day before the expected subsequent menses (cf. 41). The expected date of the subsequent menses onset was validated by a chromatographic ovulation testing phase around the expected date of ovulation using Femometer^®^ LH ovulation rapid test strips with a corresponding smart app with an intelligent interpretation function (Femometer^®^ app). This ovulation testing phase lasted until receiving a positive result. In case of persistent negative or invalid testing results over 10 days, we asked the participants to repeat the ovulation testing during the next cycle. In these cases, the AA of the late-luteal phase was postponed to the next cycle. We provided constant technical support regarding the study procedure during the entire study via phone, e-mail or online-meetings.

To prevent sequence effects, the AA-start was randomized between the follicular and the late luteal phase. In burst 1, 32 women and in burst 2, 28 started their AA during the follicular phase. During the AA-phases, participants were instructed to wake up no later than 8:00 a.m. At each assessment point at semi-random times with inter-assessments intervals of 45 to 145 min, starting at a fixed time of 9:00 am and ending approximately at 9:30 pm, they were asked to rate their momentary affect and stress-appraisal since the last prompt (or during the last 1.5 hours at the first assessment). Participants could reject or postpone prompts for up to 15 min. Rejected or ignored signals were coded as missings. With a time lag of 10 min after the completion of subjective assessments, participants were prompted to collect saliva samples.

##### 2.2.2.1 Subjective AA-measures

At each assessment, momentary affect was assessed using 12 items, which were derived from the PANAS (42) and previous AA-studies (e.g., 31, 43). The participants were asked to indicate the extent to which they experienced negative affect (NA; i.e., felt *upset*, *irritated*, *nervous*, *listless*, *down*, and *bored)* and positive affect (PA; i.e., felt *cheerful*, *energetic*, *enthusiastic*, *satisfied*, *relaxed*, and *calm)* on a 7-point Likert scale ranging from 1 (not at all) to 7 (very much). For NA and PA scores, means of the respective subscales for each assessment time point were calculated.

For the assessment of stress appraisal, the participants were instructed to think about the most important event since the last prompt (or the last 1.5 hours at the first assessment of the day) and to indicate how stressful they perceived the respective event on a 7-point Likert scale ranging from 1 (not at all) to 7 (very much).

##### 2.2.2.2 Sleep

At the first assessment time at 09:00 a.m., time of awakening, sleep duration in hours and sleep quality (‘*How did you sleep last night?*’ 7-point Likert scale: 1 [very bad] - 7 [very good]) were assessed as possible covariates of cortisol activity.

##### 2.2.2.3 Saliva Cortisol

Saliva samples were collected 10 min after each subjective assessment resulting in eight saliva samples per day. At the end of subjective assessments, participants were reminded not to eat, drink anything but water, smoke, physically exercise, and brush their teeth during the next 10 min until saliva collection (44). Directly after saliva collection, participants indicated whether they had eaten, drunk anything but water, smoked or brushed their teeth (dichotomous items yes/no each) and to what extent they had engaged in physical activity on a 7-point Likert scale (1 = not at all to 7 = very much) during the last 10 min.

Saliva samples were stored in the participants’ home freezers until return to the lab. In the lab, the samples were frozen at -20°C until the biochemical analysis at Dresden LabService GmbH, Germany. After thawing, the samples were centrifuged at 3,000 rpm for 5 min, which resulted in a clear supernatant of low viscosity. Salivary concentrations were measured using commercially available chemiluminescence immunoassay with high sensitivity (IBL International, Hamburg, Germany). The intra- and interassay coefficients for cortisol were below 9%.

### 2.3 Statistical analyses

The data consisted of momentary measurements (Level-1) within bursts (Levels-2), which were nested within individuals (Level-3), such that multilevel models (MLM) were fit.

First, cortisol raw data were log-transformed to adjust for skewness. Per burst, outliers above 3 standard deviations from the group mean were winsorized to 3 standard deviations (44, 45). Next, we checked for potential confounding effects of study day and weekday vs weekend on all outcomes (NA, PA, stress appraisal, cortisol activity). Additionally, possible confounding effects of time since first assessment were tested for subjective measures. For cortisol activity, we checked for the following additional possible confounders: saliva collection time since awakening, habitual smoking, age, sleep quality, sleep duration, as well as drinking anything but water, smoking cigarettes, eating, brushing teeth, and the level of physical activity during the last 10 min before saliva collection. If significant, these possible covariates were retained in the models (*p* < 0.05). This applied to assessment day for all outcomes. For stress appraisal, weekday was retained as an additional covariate in the respective models. For cortisol activity, additional covariates were saliva collection time since awakening, weekday vs. weekend, sleep duration and smoking during the last 10 min. Furthermore, the Level-3 predictors *childhood adversity* and *stressful life events* were grandmean-centered. For statistical purposes, the burst variable (1 vs 2) was recoded as (0 vs 1).

In general, the statistical analyses were performed through three steps. First, we estimated main effects of cycle phase (0 = follicular phase vs 1 = late luteal phase), childhood adversity and recent stressful life events on each outcome in separate MLMs (models 1). Next, the main effects of *burst* (0 = burst 1 vs 1 = burst 2) and the interaction term of cycle phase * burst were added to these models in order to investigate whether the effects of cycle phase on daily outcomes would be stable over bursts (models 2). Models 2 estimating burst related effects were rerun by including the difference score of premenstrual symptom severity between burst1 and burst 2 (PSST-scores during the late luteal phase) as a covariate in order to control for possible premenstrual symptom alterations over time.

In a third step, these models were expanded by entering the interaction term of childhood adversity with cycle phase to assess the impact of childhood adversity on the cyclicity of daily outcomes (models 3). In case of significant interaction effects, we subsequently estimated simple effects for significant interaction terms. Additionally, we examined possible age-related differences in cycle-specific effects of childhood adversity on mood and cortisol activity by adding age as a main factor and childhood adversity * age * cycle phase as an interaction term to respective models.

The main analyses were performed in R (46), using the lmer functions from the package lme4 and lmerTest (47, 48). Due to numeric limitations of the software R, simple effects for models 2 and 3 were estimated via IBM SPSS Statistics Version 28 (49).

A repeated measures ANOVA was performed to compare the PSST-scores between burst 1 and burst 2 during the follicular and the late luteal phase.

## 3 Results

### 3.1 Descriptives

**Table 1** shows descriptives on demographics and clinical characteristics of the current sample. The repeated-measures ANOVA showed that PSST scores differed significantly across cycle phases and bursts (*F* (2, 85) = 140.901, *p* <.001). *Post hoc* pairwise comparisons showed that PSST-scores during the follicular phase did not differ between burst 1 and burst 2 (mean difference = -0.667, *SE* = 1.209, *p* = .584). However, the participants showed lower PSST-scores during the luteal phase in burst 2 compared to burst 1 (mean difference = -3.375,*SE* = 1.129, *p = .*004), indicating that premenstrual symptoms weakened over the time interval of five months. Burst-specific descriptives and bivariate correlations of AA-variables, childhood adversity and stressful life events are provided in **Table 2**. At the between- and within-subject level, most variables showed negligible to moderate bivariate correlations, except for the within-subject correlation of NA and PA being highly negative. **Table 2** also includes intraclass correlation coefficients (ICC) of AA-variables. Variance decomposition using the ICC showed that 66% to 85% of the total variance in AA-variables could be attributed to within-person variations (for ICC per variable, see **Table 2**). During the AA-phase in burst 1, the compliance rate regarding subjective assessments amounted to 93.0%, and to 88.8% in burst 2, reflecting high levels of compliance (cf. 50). The AA compliance rate for cortisol sampling amounted to 92.9% in burst 1 and to 87.4% in burst 2.

**Table 1:** Demographic and clinical characteristics.

	M (*SD*)	n (%)
Demographic Variables at Burst 1		
Age	30.54 (5.59)	
Education (% with high school degree)		43 (82.7%)
Children (%)		15 (28.8%)
BMI	22.61 (3.36)	
Psychotherapy		9 (17.3%)
SSRI intake		0 (0%)
Hormonal medication intake		0 (0%)
Relationship status (% in a relationship)		25 (48.1%)
Burst 1		
Cycle length during AA	28.60 (3.47)	
PSST during Follicular Phase	10.41 (9.40)	
PSST during Luteal Phase	37.50 (9.79)	
Burst 2		
Psychotherapy start after burst 1		4 (7.7%)
SSRI intake start after burst 1		1 (1.9%)
Hormonal medication intake after burst 1		0 (0%)
Cycle length during AA	28.00 (2.34)	
PSST during Follicular Phase	10.83 (11.21)	
PSST during Luteal Phase	34.66 (11.01)	

BMI, Body Mass Index; AA, Ambulatory Assessment; PSST, Premenstrual Symptoms Screening Tool; SSRI, Selective Serotonin Reuptake Inhibitor. PSST scores represent sum scores.

**Table 2:** Descriptives, correlations, variability statistics of momentary outcomes and level-3 predictors.

		Bivariate Correlations						Descriptives		
Burst	Variable	1	2	3	4	5	6	M	SD_B-S_	SD_W-S_
1	1. Negative Affect	1	-0.55	0.39	0.10	0.20	0.18	2.90	0.49	1.02
	2. Positive Affect	-0.85	1	-0.46	-0.08	-0.24	-0.27	4.12	0.47	1.12
	3. Stress appraisal	0.38	-0.38	1	-0.18	0.13	0.05	0.73	0.73	1.44
	4. Cortisol ^a^	0.05	-0.03	0.06	1	-0.08	0.09	1.78	0.34	0.81
	5. CTQ	–	–	–	–	1	0.22	42.07	12.78	–
	6. SLE	–	–	–	–	–	1	2.64	1.70	–
2	1. Negative Affect	1	-0.67	0.33	0.17	0.06	0.23	2.80	0.60	0.88
	2. Positive Affect	-0.82	1	-0.27	-0.09	-0.06	-0.27	4.13	0.62	1.00
	3. Stress appraisal	0.25	-0.26	1	-0.24	-0.09	0.02	2.17	0.71	1.22
	4. Cortisol	0.04	0.00	0.04	1	0.03	0.08	1.78	0.37	0.77
	5. CTQ	–	–	–	–	–	0.24	–	–	–
	6. SLE	–	–	–	–	–	1	–	–	–
	ICCs across Bursts									
	ICC_Personlevel_	0.18	0.17	0.17	0.09	–	–	–	–	–
	ICC_Burstlevel_	0.06	0.17	0.05	0.06	–	–	–	–	–

CTQ, Childhood Trauma Questionnaire; SLE, Stressful Life Events; ICC, Intraclass Correlation Coefficient; SD_B-S_, Between-subject standard deviation; SD_W-S_, Within-subject standard deviation. Between-subject correlations are presented above the diagonal; within-subject correlations among momentary measures are presented below the diagonal. Given that CTQ and SLE represent single time-point scores as cross-sectional data, no within-subject correlations between trait and state measures could be computed. Means and between-subject standard deviations were calculated based on aggregated person-mean scores.

a Cortisol data were log-transformed and winsorized to three standard deviations of the sample mean.

Participants who were interviewed in person and those interviewed online via RedConnect did not statistically differ in their age (*t* (50) = 1.45, *p* = .155), the number of their PMDD symptoms as assessed with SCID-PMDD (30) (*t* (50) = -0.45, *p* = .655) and in their PSST-sumscores (*t*(50) = -0.17, *p* = .870) at baseline.

### 3.2 Effects of cycle phase and burst

Main results are presented in models 1 of **Tables 3**, **4**. Across bursts, cycle phase was significantly associated with all subjective outcomes (i.e., NA, PA, stress appraisal), but not with cortisol activity (*F*(1, 5538) = 0.612, *p* = .805). In particular, women with PMDD showed higher NA (*F*(1, 5817) = 2005.917, *p* <.001), lower PA (*F*(1, 5825) = 1939.921, *p* <.001) and higher stress appraisal (*F*(1, 5820) = 53.627, *p* <.001) during the late luteal phase compared to the follicular phase (see respective models 1 in **Tables 3**, **4**). In contrast, no main effects of childhood adversity were identified on NA (*F*(1, 101) = 0.890, *p* = .347), PA (F(1, 102) = 0.819, *p* = .368), stress appraisal (*F*(1, 102) = 0.056, *p* = .813) and cortisol activity (*F*(1, 103) = 0.053, *p* = .818). Recent stress life events were associated with lower momentary PA (*F*(1, 102) = 5.850, *p* = .017; see model 1 in **Table 3**), with no main effects on NA (*F*(1, 101) = 2.754, *p* = .100), stress appraisal (*F*(1, 102) = 0.027, *p* = .869) and cortisol activitiy (*F*(1, 101) = 0.167, *p* = .684).

**Table 3:** Multilevel Analyses of cycle phase, childhood adversity and burst on negative affect and positive affect.

	Negative Affect						Positive Affect					
	Model 1		Model 2		Model 3		Model 1		Model 2		Model 3	
	*B*	SE	*B*	SE	*B*	SE	*B*	SE	*B*	SE	*B*	SE
Fixed Effects												
Intercepts	2.351***	0.058	2.342***	0.079	2.338***	0.080	4.660***	0.058	4.711***	0.079	4.717***	0.079
Study day	0.003	0.005	0.003	0.005	0.003	0.005	0.004	0.005	0.004	0.005	0.003	0.005
Cycle^a^	0.986***	0.022	1.100***	0.030	1.099***	0.030	- 1.085***	0.025	- 1.234***	0.034	- 1.234***	0.035
SLE	0.054	0.032	0.054	0.032	0.0530	0.033	-0.077*	0.032	-0.077*	0.032	- 0.076*	0.032
CTQ	0.004	0.004	0.004	0.004	-0.001	0.004	-0.038	0.042	-0.004	0.004	0.004	0.004
Burst^b^			0.028	0.109	0.030	0.110			- 0.114	0.111	- 0.118	0.108
Cycle x Burst			-0.238***	0.043	-0.236***	0.043			0.313***	0.048	0.310***	0.048
Cycle x CTQ					0.010***	0.002					-0.015***	0.002

CTQ, Childhood Trauma Questionnaire; SLE, Stressful Life Events.

* p <.05, ** p <.01, *** p <.001.

a Reference category = Follicular phase.

b Reference category = Burst 1.

**Table 4:** Multilevel analyses of cycle phase, childhood adversity and burst on stress appraisal and cortisol activity.

	Stress Appraisal						Cortisol^c^					
	Model 1		Model 2		Model 3		Model 1		Model 2		Model 3	
	*B*	SE	*B*	SE	*B*	SE	*B*	SE	*B*	SE	*B*	SE
Fixed Effects												
Intercepts	2.486***	0.082	2.538***	0.109	2.533***	0.110	3.086***	0.082	3.075***	0.088	3.083***	0.088
Time	—		—		—		-0.138***	0.002	-0.138***	0.002	-0.138***	0.002
Weekday	-0.136**	0.042	-0.137***	0.042	-0.133**	0.041	-0.037*	0.018	-0.037*	0.018	-0.038*	0.018
Study day	-0.060***	0.008	-0.060***	0.008	-0.060***	0.008	0.010**	0.003	0.010**	0.003	0.010**	0.003
Smoking	—		—		—		0.283	0.154	0.283	0.154	0.292	0.154
Sleep duration	—		—		—		-0.030**	0.011	-0.030**	0.011	-0.031**	0.011
Cycle^a^	0.262***	0.036	0.431***	0.049	0.431***	0.049	-0.004	0.011	0.015	0.021	0.015	0.021
SLE	0.007	0.044	0.008	0.044	0.007	0.044	0.008	0.019	0.007	0.002	0.008	0.019
CTQ	0.001	0.006	0.001	0.005	-0.004	0.006	-0.001	0.003	-0.001	0.002	0.001	0.003
Burst^b^			- 0.090	0.148	-0.087	0.149			0.029	0.066	0.028	0.066
Cycle x Burst			- 0.354***	0.070	-0.353***	0.007			-0.041	0.031	-0.042	0.031
Cycle x CTQ					0.011***	0.003					-0.003*	0.001

CTQ, Childhood Trauma Questionnaire; SLE, Stressful Life Events. The covariate time represents time of saliva collection since awakening.

* p <.05, ** p <.01, *** p <.001.

a Reference category = Follicular phase.

b Reference category = Burst 1.

c Cortisol data were log-transformed and winsorized to three standard deviations of the sample mean.

Next, longer-term cycle-phase-specific variations in daily life outcomes were investigated (see respective models 2 in **Tables 3**, **4**). Burst significantly moderated the effects of cycle phase on NA (*F*(1, 5812) = 30.250, *p* <.001), PA (*F*(1, 5820) = 41.845, *p* <.001) and stress appraisal (*F*(1, 5814) = 25.497, *p* <.001), but not on cortisol activity (*F*(1, 5532) = 1.739, *p* = .187). In both bursts, NA (mean difference _Burst-1 = _1.099, *SE*_Burst-1 = _0.030, *p* <.001; mean difference _Burst-2 = _0.861, *SE*_Burst-2 = _0.031, *p* <.001) was higher and PA (mean difference _Burst-1_ = -1.235, *SE*_Burst-1 = _0.034, *p* <.001; mean difference _Burst-2_ = -0.921, *SE*_Burst-2 = _0.035, *p* <.001) was lower during the late luteal phase compared to the follicular phase, with smaller cycle phase differences in burst 2 compared to burst 1. Stress appraisal in daily life was higher during the late luteal phase compared to the follicular phase only in burst 1 (mean difference _Burst-1 = _0.430, *SE*_Burst-1 = _0.049, *p* <.001) but not in burst 2 (mean difference _Burst-2 = _0.079, *SE*_Burst-2 = _0.051, *p* = .122). In sum, these results indicate that deteriorations in momentary mood from the follicular to the late luteal phase weakened over the 5-months period but were still significant, whereas the premenstrual increase in stress appraisal was no longer apparent in burst 2. Cortisol activity, in turn, showed no variations across cycle phases and bursts.

After including the PSST difference scores of the late luteal phase between burst 1 and burst 2, the interaction effects of cycle phase and burst in models 2 on all outcomes remained unchanged (NA: *b* = -0.246, *SE* = 0.044, *p* <.001; PA: *b* = 0.324, *SE* = 0.049, *p* <.001; stress appraisal: *b* = -0.312, *SE* = 0.071, *p* <.001; cortisol: *b* = -0.015, *SE* = 0.031, *p* = .628).

### 3.3 Cycle-specific associations of childhood adversity and daily variables

**Figure 1:**
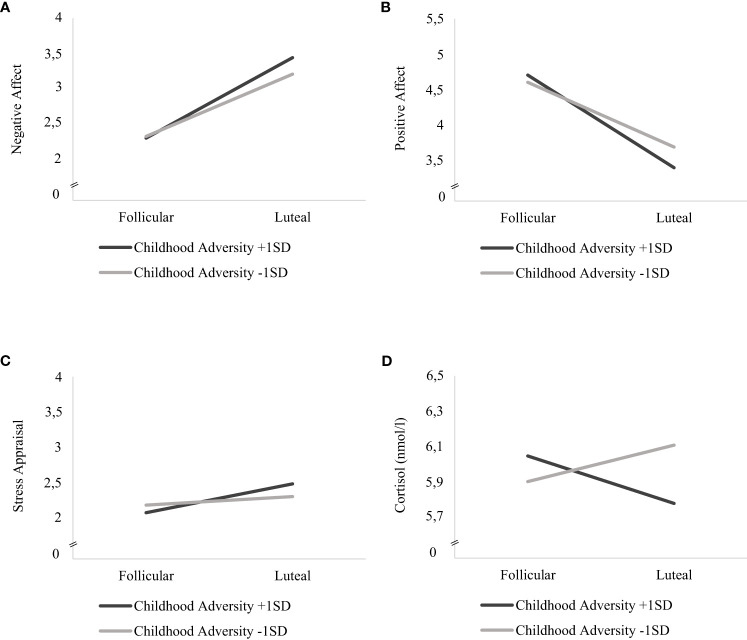
Interaction effects of childhood adversity and cycle phase on mood, stress appraisal and cortisol activity in daily life. Estimated mean values of momentary **(A)** negative affect, **(B)** positive affect **(C)** stress appraisal and **(D)** cortisol activity (nmol/l) during the follicular and late-luteal phase for high (+ 1SD) and low (- 1SD) childhood adversity.

Childhood adversity moderated the effects of cycle phase on all momentary outcomes across bursts, with significant interaction effects of *cycle phase* * *childhood adversity* on NA (*F*(1, 5811) = 33.450, *p* <.001), PA (*F*(1, 5818) = 63.428, *p* <.001), stress appraisal (*F*(1, 5813) = 15.477, *p* <.001) and cortisol activity (*F*(1, 5530) = 6.171, *p* = .013) (see respective models 3 in **Tables 3**, **4**). As can be seen in **Figures 1A–C**, women with higher childhood adversity showed higher increases in NA (mean difference = 1.106, *SE* = 0.031, *p* <.001) and stress appraisal (*mean difference* = 0.395, *SE* = 0.050, *p* <.001) as well as higher decreases in PA (mean difference = -1.269, *SE* = 0.034, *p* <.001) from the follicular to the late luteal phase compared to women with lower childhood adversity (NA: mean difference_NA_ = 0.855, *SE*_NA_ = 0.031, *p*_NA_ <.001; stress appraisal: mean difference_stress_ = 0.115, *SE*_stress_ = 0.050, *p*_stress_ = .021; PA: mean difference = -0.887, *SE*_PA_ = 0.034, *p*_PA_ <.001). Regarding cortisol activity, women with higher childhood adversity showed lower basal cortisol activity during the late luteal phase compared to the follicular phase (*mean difference* = -0.044, *SE* = 0.022, *p* = .048) whereas women with lower childhood adversity did not exhibit cycle-phase-specific variations in cortisol activity (mean difference = -0.033, *SE* = 0.022, *p* = .128; see **Figure 1D**).

In addition, we examined possible age-related differences in cycle-specific effects of childhood adversity on mood and cortisol activity. The respective interaction analyses of childhood adversity * age * cycle phase revealed no significant effects on NA (*F*(1, 5806) = 0.512, *p* = 0.474), PA (*F*(1, 5811) = 1.691, *p* = 0.193), stress appraisal (*F*(1, 5806) = 2.90, *p* = 0.089) or cortisol activity (*F*(1, 5522) = 0.362, *p* = 0.574).

## 4 Discussion

Traumatic events during lifetime are prevalent in women with PMDD and predict higher premenstrual symptom levels (18, 22). Given that especially childhood adversity interferes with sensitive developmental periods regarding affective-cognitive and neuroendocrine processes, the present study focused on the role of childhood adversity for menstrual cycle related variations of mood, stress appraisal and cortisol during daily life in women with PMDD. In the current study with two waves (bursts) of intensive AA-periods, which were separated by a 5-month interval, women with PMDD showed premenstrual worsening of positive and negative affect as well as stress appraisal across bursts, with weakening effects of cycle phase from burst 1 to burst 2. In contrast, no cyclicity of cortisol release was identified across bursts in the total sample, and the lack of cortisol cyclicity remained stable over time. By controlling for recent stressful life events, higher childhood adversity was linked to stronger mood worsening and to a stronger increase in stress appraisal from the follicular to the late luteal phase. Childhood adversity also moderated cortisol cyclicity. Women with higher childhood adversity exhibited lower cortisol levels during the late luteal compared to the follicular phase, whereas no such cyclicity was found in women with lower childhood adversity.

### 4.1 Effects of cycle phase and time

Consistent with findings from a previous AA-study (43), negative affect during daily life increased and positive affect decreased from the follicular to the late luteal phase in the current sample, thereby mirroring the clinical presentation of PMDD characterized by key premenstrual affective symptoms (6). Furthermore, previous research has shown that women with severe premenstrual symptoms generally report higher perceived chronic stress compared to healthy controls (cf. 51). In line with Beddig et al. (15), the current study further shows that in women with PMDD, perceived stress in daily life appears to be particularly high during the late luteal phase. Regarding cortisol activity, no such cyclicity was identified in the current total sample, which was, however, moderated by childhood adversity (see below).

Over a period of five months, the premenstrual increase in negative affect and the decrease in positive affect weakened, while the effect of cycle phase was still significant. In general, fluctuations of premenstrual symptoms over time have to be considered (cf. 52), and a previous population-based cohort study showed that only 46% of women diagnosed with severe PMS at baseline qualified for severe PMS one year later, whereas 19% qualified for moderate PMS and further 35% reported premenstrual symptom and impairment levels no more qualifying for the condition (53). Similarly, Hart et al. (54) demonstrated that prospective scores in premenstrual symptoms from one cycle predicted only 14% of the variance in the next cycle, pointing to inter-cycle variability in premenstrual symptoms.

The premenstrual increase in stress appraisal, in turn, was no longer observed at burst 2 in the present study. One possible reason for the diminished cyclicity in stress appraisal together with reduced premenstrual symptoms (PSST) over time might be that for the majority of participants, the baseline-interview at burst 1 was the first time when they were validated and recognized for their PMDD related distress. This might have led to an increased awareness and accepting attitude toward their premenstrual symptoms or to behavioral life-style changes to cope with them over time. It seems noteworthy that four women started psychotherapy and one woman started pharmacotherapy with a Selective Serotonin Reuptake Inhibitor after burst 1, indicating possible increased awareness and active coping. However, these explanations are highly speculative, and more research systematically investigating protective factors against the chronification of mood and stress cyclicity in PMDD is warranted. Furthermore, it is to note that the observed weakening of premenstrual mood worsening and stress appraisal from burst 1 to burst 2 could not be sufficiently explained by the observed parallel improvement in premenstrual symptoms, as assessed by the PSST. Thus, repeated assessments of mood and stress perception during daily life may yield a more fine-graded picture of the cyclicity of subjective experiences and their fluctuations over time than difference scores in premenstrual symptoms as assessed retrospectively at the end of the luteal phase (cf. 55).

### 4.2 Effects of childhood adversity

Research on childhood adversity indicates that childhood adversity is associated with higher negative affect, lower positive affect (56, 57) and higher perceived stress (58) during daily life in adult non-clinical samples, and with the development of various forms of psychopathology (cf. 26, 59). In these various contexts, researchers generally investigated and identified main effects of childhood adversity on respective outcomes. However, childhood adversity did not exert any main effects on mood and stress appraisal in daily life, when investigated across the total cycle, in the present sample. In contrast, our results suggest that childhood adversity appears to specifically impact the *cyclicity* of daily life experiences in women with PMDD. As hypothesized, childhood adversity was associated with stronger increases in negative affect and stronger decreases in positive affect from the follicular toward the late luteal phase. These findings align with initial evidence that lifetime trauma and recent stressful life events increase the severity of premenstrual symptoms in women with severe PMS and PMDD (9), thereby indicating that childhood adversity represents a distal risk factor for severe clinical manifestations of PMDD. The current findings additionally refine previous findings by disentangling childhood and adulthood adversity, thereby focusing on the net-effects of childhood adversity. The present study also demonstrates that the premenstrual increase in daily stress appraisal is particularly strong in women with higher childhood adversity. Thus, childhood adversity appears to render women with PMDD even more sensitive to daily life stress, especially during their vulnerable late luteal phase.

In parallel to mood and stress appraisal, childhood adversity did not exert a general main effect on cortisol activity during daily life in the present sample when cumulated across the cycle. This is again different to demonstrated main effects of childhood adversity on cortisol when samples with other psychopathologies are investigated. Here, a majority of studies show that childhood adversity is associated with lower resting cortisol activity and blunted cortisol reactivity in clinical and non-clinical samples (26, 59, 60).

In contrast to these studies, and similar to the current subjective outcomes, childhood adversity specifically affected the *cyclicity* of cortisol, thereby supporting the assumption of an interaction of adversity related HPA axis alterations and the HPG-axis in PMDD (cf. 18, 22). In particular, women with higher childhood adversity displayed cortisol cyclicity with lower levels during the late luteal phase compared to the follicular phase, while no such cyclicity was identified in women with lower childhood adversity. Placing these results in the context of previous findings, the study by Girdler et al. (12) found that, regardless of cycle phase and abuse history, women with PMDD showed significantly lower resting baseline plasma cortisol levels compared to controls. Furthermore, prior abuse was not associated with altered cortisol reactivity in response to mental stress (12). In contrast, Girdler et al. (25) reported a trend for lower cortisol levels in abused compared to non-abused women, regardless of PMDD status, whereas in both studies (12, 25), abused women with PMDD specifically displayed higher adrenergic activity at rest and during stress. However, our studies are not completely comparable, since we assessed saliva cortisol during daily life and not plasma cortisol before and during stress.

The observed cortisol cyclicity with lower levels during the late luteal phase in women with higher childhood adversity should also be discussed in light of the two recent meta-analyses in healthy women (16, 17). Both meta-analyses identified menstrual cycle related cortisol variation with lower levels of cortisol during the luteal compared to the follicular phase, suggesting that this pattern may be an adaptive response to increasing ALLO levels in the luteal phase. ALLO is a neurosteroid metabolit of progesterone and a positive allosteric modulator of the GABAA receptor in the brain, thereby potentiating the anxiolytic and sedative effects of GABA, which plays a critical role in negative modulation of the HPA axis (cf. 8, 61). However, a growing body of evidence suggests that women with PMDD show a paradoxical reaction toward the fluctuating ALLO levels during the luteal phase with typically increased premenstrual irritability and other affective core symptoms (e.g., 3, 62).

In our previous analysis from the present project with data from burst 1 (13), we identified cortisol cyclicity with lower levels during the late luteal phase in a sample of healthy women (cf. 16, 17). In contrast, no such cyclicity was found in the latter total sample of women with PMDD, together with overall decreased cortisol levels in the PMDD sample compared to healthy controls. The present analysis replicates the lack of cortisol cyclicity for the total PMDD sample, now combined for burst 1 and burst 2 (cortisol data for healthy women were collected only at burst 1). However, when considering the moderator effect of childhood adversity, we observed a further decrease during the late luteal phase in those women with higher childhood abuse, pointing to a possible specific “ecophenotype” (59) in PMDD. Such childhood adversity related ecophenotypes, stemming from the environmental experience of high childhood adversity and their consequences, have been identified for individuals with a variety of mental disorders, which are clinically and neurobiologically distinct from those with low exposure. These differences include abnormal HPA axis activity and autonomic responses to stressors (59). The particularly low luteal cortisol levels in women with high childhood adversity may therefore indicate a unique ecophenotype of PMDD which, in turn, contributes to the observed aggravating effects of childhood adversity on premenstrual mood and stress appraisal. While these conclusions are still highly speculative, the search for adversity related ecophenotypes in PMDD as a cyclic disorder appear highly promising since they are directly implicated in the interaction of early adversity with the HPA and the HPG axes (22). However, more detailed future research is clearly warranted aiming at elucidating possible childhood adversity related PMDD subtypes with respect to their molecular and physiological consequences, not least in order to be able to offer more individualized treatment options to affected women.

### 4.3 Clinical implications

In sum, our results imply that women with PMDD and higher childhood adversity differ from those with lower childhood adversity with respect to their cyclical course of mood, stress appraisal and cortisol release during daily life. In particular, childhood adversity appears to increase the risk for premenstrual affective and cognitive deteriorations and neuroendocrinological vulnerabilities, which in turn may predict a worse clinical course and a poorer treatment response, as observed in other psychiatric disorders (e.g., 1, 59). Therefore, the potential impact of childhood adversity on the clinical course of PMDD needs to be considered in future research. For example, pharmacological research, which currently mainly focuses on novel agents aiming at stabilizing the ALLO level signaling during the luteal phase (for reviews, see 61, 63), should investigate whether childhood adversity moderates the efficacy of these drugs. In psychotherapy research, a similar consideration should be given to possible early adversity related affective-cognitive dysregulations. Notably, childhood adversity is linked to a higher rumination tendency in adulthood, which in turn is associated with worse clinical outcomes (64). Women with PMDD exhibit higher habitual rumination compared to healthy controls (e.g., 31) and higher momentary rumination during the late luteal phase compared to the follicular phase (15), which in turn predicts higher negative affect specifically during the late luteal phase (43). Thus, rumination might represent a psychological mechanism by which childhood adversity is linked to higher premenstrual mood worsening, and future studies should take childhood adversity into account in interventions addressing rumination. Furthermore, the inclusion of childhood adversity related treatment components could be investigated in clinical trials, for example by targeting adversity related core beliefs or specific stress management skills in affected women with PMDD.

In general, a more systematic consideration of potential childhood adversity in PMDD research and treatment is clearly warranted, and childhood adversity should be regularly assessed in the clinical care of women with PMDD. The identification of possible targeted and individualized treatment options for affected women will hopefully improve the hitherto only modest treatment achievements in PMDD (cf. 65), and will also prevent premature treatment termination due to weak therapeutic alliance, as already discussed in the context of other mental disorders (cf. 66).

### 4.4 Strengths and limitations

This is the first study investigating associations of childhood adversity with momentary mood, stress appraisal and cortisol activity across the menstrual cycle in women with PMDD using an intensive AA design with two measurement bursts. In order to rule out possible effects of psychiatric comorbidities and pharmacology, we used strict exclusion criteria such as the presence of a current depressive episode as well as the intake of psychopharmacology and drugs affecting the HPA axis. We further controlled for recent stressful life events. Thus, the current findings represent unique effects of childhood adversity on cycle related mood, stress appraisal and cortisol in women with PMDD. Another strength is the use of a chromatographic ovulation test for the validation of ovulatory cycles.

The present study also has some limitations. First, we decided not to administer prospective symptom ratings over two cycles, as required by DSM-5 for a definite diagnosis of PMDD (6), in order to avoid overburdening participants within the extensive AA-design with two bursts. Instead, provisional PMDD diagnoses were made using a structured and validated diagnostic interview (SCID-PMDD) (30). Moreover, with *N* = 52, our sample size was generally moderate. However, considering the number of Level 3 units (participants) and Level 1 units (observations: 2 bursts * 8 assessments days * 8 assessments per day) in our AA-design, the power was expected to reach the threshold of 80% to detect small to medium effect sizes (67). Furthermore, although adversity during the entire childhood has been shown to be linked to the development of psychopathology, the duration, timing, and developmental stage during exposure to childhood adversity may lead to differential effects regarding psychological and neuroendocrine outcomes (cf. 2, 28). Future research delineating specific critical developmental windows during which childhood adversity specifically influences the HPA-HPG interaction and has the most clinical impact on premenstrual symptoms is warranted. Similarly, not only recent life events during the past 12 months but also traumas and major stressful life events at different life stages during the entire adulthood period might be relevant for the extent of premenstrual symptoms. Thus, while our analyses controlled for the potential confounding recency effects of experiences during the past year, future larger prospective studies could benefit from systematic life-stage-related assessments of stressful events.

Moreover, childhood adversity was assessed retrospectively, bearing the risk for recall bias and pointing to the need for prospective cohort studies in this context. This would also allow to investigate the effects of childhood adversity on the risk of developing PMDD, and to contribute to the investigation of transitions from PMS to a full syndrome PMDD. Regarding the longer-term effects of childhood adversity on the cyclicity of mood, stress appraisal and cortisol, longer time intervals between bursts might be suitable to uncover clinically relevant and potentially slower contextual processes such as life transitions or marked clinical changes (e.g., 68).

## 5 Conclusions

In conclusion, high levels of experienced childhood adversity appear to predict more pronounced premenstrual mood worsening and higher premenstrual increase of stress appraisal during daily life in women with PMDD. Women with higher childhood adversity further seem to differ from those with lower childhood adversity also in terms of saliva cortisol cyclicity by showing lower cortisol levels during the late luteal phase, possibly indicating a specific ecophenotype of PMDD. These identified affective-cognitive and neuroendocrine effects of childhood adversity in women with PMDD underscore the need for further research to delineate possible subgroups in PMDD. Understanding these distinctions can lead to more personalized interventions for women with PMDD, taking into account their unique experiences of childhood adversity.

## Data availability statement

The raw data supporting the conclusions of this article will be made available by the authors, without undue reservation.

## Ethics statement

The studies involving humans were approved by Ethics Committee of the Medical Faculty Mannheim, Heidelberg University (institution grant approval 2015-572N-MA). The studies were conducted in accordance with the local legislation and institutional requirements. The participants provided their written informed consent to participate in this study.

## Author contributions

SN: Conceptualization, Data curation, Formal Analysis, Investigation, Methodology, Writing – original draft, Writing – review & editing. IS: Data curation, Investigation, Writing – review & editing. IR: Supervision, Writing – review & editing. CK: Conceptualization, Funding acquisition, Methodology, Supervision, Writing – review & editing.
